# The Links Between Disability, Incarceration, And Social Exclusion

**DOI:** 10.1377/hlthaff.2022.00495

**Published:** 2022-10

**Authors:** Laurin Bixby, Stacey Bevan, Courtney Boen

**Affiliations:** University of Pennsylvania, Philadelphia, Pennsylvania.; University of Pennsylvania.; University of Pennsylvania.

## Abstract

Disabled people are disproportionately incarcerated and segregated from society through a variety of institutions. Still, the links between disability and incarceration are underexplored, limiting understanding of how carceral institutions punish and contribute to the social exclusion of disabled people. Using data from the 2016 Survey of Prison Inmates, we estimated disability prevalence in state and federal prisons, assessing disparities by race, ethnicity, and sex, and we examined inequities in previous residence in other “punitive” and “therapeutic” institutions. Sixty-six percent of incarcerated people self-reported a disability, with Black, Hispanic, and multiracial disabled men especially overrepresented in prisons. Compared with nondisabled incarcerated people, disabled incarcerated people were more likely to have previously resided in other institutions, such as juvenile detention facilities and psychiatric hospitals. Together, our findings advance the understanding of disability in carceral institutions, highlighting the need for policy interventions redressing the mechanisms contributing to the high incarceration risks of disabled people and the disabling nature of prisons and other carceral institutions.

The expansion of mass incarceration during the past sixty years in the US is unprecedented. With more than 1.2 million people incarcerated in prisons as of December 2021, the US is a global leader in incarceration.^1^ Importantly, disability prevalence has been found to be higher among incarcerated compared with nonincarcerated people.^2^ Psychiatric disability has been shown to be especially common among currently and formerly incarcerated people,^3^ and estimates from the 2004 Survey of Inmates in State and Federal Correctional Facilities found that more than 40 percent of incarcerated people reported nonpsychiatric disability.^2^ This high prevalence reflects the reality that disabled people^4^ are at high risk for incarceration and that carceral institutions are disabling.^5^ The labeling and treatment of disabled people by medical and judicial institutions has historically been intertwined with notions of social danger, deviance, and criminality, resulting in the disproportionate confinement of disabled people in incarcerating spaces such as prisons, jails, and psychiatric hospitals.^6–9^ Further, prisons and other carceral institutions are characterized by high levels of stress, fear, social isolation, infectious disease, and violence exposure,^10,11^ all of which can increase disability risks.

Despite growing evidence of the links between disability and incarceration, two gaps warrant attention. The first is the lack of updated estimates of disability prevalence in prisons, with consideration of both psychiatric and nonpsychiatric disabilities, as well as estimates by race, ethnicity, and sex. A large body of research documents inequities in incarceration by race and ethnicity, with Black men facing particularly high levels of imprisonment.^12,13^ Although men make up a disproportionate share of incarcerated people, the number of women incarcerated in prisons and jails has increased by more than 475 percent during the past four decades, going from 26,326 women in 1980 to 152,854 women in 2020.^14^ These patterns highlight the need for studies of disability prevalence among incarcerated people to consider disparities by race, ethnicity, and sex.

Second, little is known about how disabled incarcerated people interface with other institutions in ways that shape their lives and well-being before, during, and after prison incarceration. Prisons are not the only sites where carceral principles, supervision, and control are exercised. Although not all the same, other types of institutions, including both punitive and traditionally considered “therapeutic” institutions, also disproportionately confine, segregate, and punish disabled people.^6–9^ Inattention to these institutions limits understanding of their role in shaping the lives of disabled incarcerated people.

Using data from the 2016 Survey of Prison Inmates (SPI),^15^ this study had two aims. First, we obtained detailed descriptive evidence of the prevalence of disability—including psychiatric and nonpsychiatric disability—in state and federal prisons, paying particular attention to the intersections of disability, race, ethnicity, and sex. Second, we estimated the percentage of people incarcerated in state or federal prison who reported previous residence in other institutions, including both punitive and therapeutic institutions. By assessing disability prevalence in prisons and documenting disparities in previous institutional residence by disability, race, ethnicity, and sex, this study provides new insights into the institutions that disproportionately punish and isolate disabled people, with important implications for policy and intervention. Taken together, results reveal how systemic ableism (discrimination that favors nondisabled people) is embedded in US institutions in ways that collectively shape the lives, health, and well-being of disabled people along racial and gender lines.

## Study Data And Methods

### DATA SOURCE AND ANALYTIC SAMPLE

This study used cross-sectional data from the latest iteration of the Survey of Prison Inmates. Conducted by the Bureau of Justice Statistics in 2016, the SPI used a stratified two-stage sample design to obtain data from 24,848 people in state or federal prisons ages eighteen and older.^15^ It provides national estimates of the state and federal prison population across a variety of domains, including demographic and disability measures and information about prior residence in other institutions. Given minimal missingness in the data (less than 5 percent per variable), analyses excluded respondents with missing data.^16^ The final analytic sample included 22,660 people.

### MEASURES

Our analyses focused on estimating disability prevalence in state and federal prisons. All measures were self-reported by survey respondents. We considered someone to have a psychiatric disability if they self-reported being diagnosed with any of the following: bipolar disorder, depressive disorder, schizophrenia or other psychotic disorder, posttraumatic stress disorder, anxiety disorder, personality disorder, or other mental or emotional health condition. We considered someone to have a nonpsychiatric disability if they self-reported any of the following: deaf or hard of hearing, blind or low vision, cognitive disability, mobility disability, self-care disability, independence disability, attention deficit hyperactivity disorder, learning disability, or previous enrollment in “special education.” We considered someone disabled if they self-reported any disability, either psychiatric or nonpsychiatric. See the online appendix for question wording.^17^

We also estimated previous residence in other institutions. We considered someone to have previously resided in “any institution” if they reported having ever resided in any of the following: juvenile correctional facility, local or county jail, other state or federal prison, residential treatment facility (that is, for alcohol or drug treatment), hospital (that is, hospitalized for mental health treatment), or other agency or institution (for example, mental health facility or group home). We categorized these institutions by their stated intent: punitive versus therapeutic.^7^ “Punitive” institutions included juvenile correctional facilities, jails, and prisons. “Therapeutic” institutions included residential treatment facilities, hospitals, and other agencies or institutions.

We also assessed disparities by race and ethnicity. SPI respondents were asked to self-identify their race and Hispanic ethnicity. In this study we used a constructed, five-category measure of race and ethnicity (non-Hispanic Black, Hispanic, non-Hispanic White, other single race and non-Hispanic, and multiracial and non-Hispanic) and a binary measure of sex (men and women). Supplementary analyses examined age-standardized and age-specific estimates. Results were substantively similar to the final results presented here.

### STATISTICAL ANALYSIS

We used weighted descriptive statistics to estimate disability prevalence in state and federal prisons and to describe estimates of previous residence in other institutions among incarcerated people. In our analyses we also examined disparities in disability prevalence and previous institutional residence by race, ethnicity, and sex, as well as at their intersections. We constructed 95% confidence intervals for all estimates and assessed the statistical significance of group differences, using two-tailed tests of the differences in proportions across groups where the null hypothesis was that the proportions were the same. All estimates were weighted using national-level survey weights, which weighted people by the inverse probability of selection within each sampled prison and adjust for nonresponse.

### LIMITATIONS

This study had several limitations. First, the SPI data were cross-sectional, collected periodically between 1974 and 2016. However, we were unable to assess how estimates of disability prevalence and previous institutional residence changed over time because the questions wre not consistently asked across waves. Second, the SPI’s disability measures did not include disability severity or timing of disability onset. Thus, we could not disaggregate by disability severity nor assess whether someone was disabled before entering prison. In addition, disability measures were self-reports and were not inclusive of all forms of disability. Third, we could not assess gender differences beyond men and women because of sample size constraints. Fourth, we used a combined race and ethnicity measure, but there may be heterogeneity within racial and ethnic categories that we were unable to capture. Fifth, the SPI did not include the timing or duration of previous residence in other institutions. Sixth, the SPI data were limited in scope to state and federal prisons, excluding private and for-profit prisons. Finally, the list of institutions available in the SPI data was not comprehensive of all spaces of confinement that people may have resided in before incarceration, such as Immigration and Customs Enforcement detention centers or nursing homes. Thus, our estimates of previous residence in other institutions were likely conservative.

## Study Results

### DISABILITY PREVALENCE IN STATE AND FEDERAL PRISONS

Among people in state and federal prisons in 2016, an estimated 40.4 percent reported a psychiatric disability, and 56.0 percent reported a nonpsychiatric disability (exhibit 1). Overall, an estimated 66 percent of incarcerated people were disabled. Exhibit 1 presents the distribution of specific types of disability and shows that bipolar, depressive, and anxiety disorders were especially common among psychiatric disabilities, as were cognitive disability, attention deficit hyperactivity disorder, and having been enrolled in “special education” among nonpsychiatric disabilities.

Our results underscore the urgent need to prevent and redress the disproportionate incarceration of disabled people.

There were disparities in disability by race and ethnicity (exhibit 2). Among the state and federal prison population in 2016, an estimated 58.3 percent of Black, 57.9 percent of Hispanic, 75.0 percent of White, 65.8 percent of other race, and 77.6 percent of multiracial people were disabled. Of the total state and federal prison population, approximately 42 percent were racially minoritized, disabled people (data not shown).

A higher estimated percentage of incarcerated women reported disability (79.5 percent) compared with incarcerated men (64.6 percent) (exhibit 2). Although women were more likely to be disabled than men, men made up the overwhelming majority of people in prisons as of 2016. Among people incarcerated in state and federal prisons, an estimated 93 percent were men and 7 percent were women (data not shown). Of the total state and federal prison population, an estimated 60 percent were disabled men, 33 percent were nondisabled men, 6 percent were disabled women, and 1 percent were nondisabled women (data not shown).

Our findings reveal disparities at the intersection of disability, race, ethnicity, and sex. Across all racial and ethnic groups, more women were disabled than men, with the largest sex disparities observed among Black and Hispanic people (exhibit 2). Disabled people made up a larger proportion of the state and federal prison population than nondisabled people within all race, ethnicity, and sex groups. See the appendix for further detail on the composition of the 2016 state and federal prison population by disability, race, ethnicity, and sex.^17^

### PREVIOUS RESIDENCE IN OTHER INSTITUTIONS BY DISABILITY STATUS

Before their current incarceration, 83.4 percent of people in state and federal prisons reported previously residing in another institution (exhibit 3). An estimated 78.7 percent of those in state and federal prisons reported previous residence in a punitive institution (that is, juvenile correctional facilities, jails, and prisons), and an estimated 37.2 percent reported previous residence in a therapeutic institution (that is, a residential treatment facility, hospital, or other institution).

Still, the experience of residing in other institutions was highly unequal, with disabled people more likely than nondisabled people to report previous residence in another institution. This pattern held across all institution types. Although disabled people were only slightly more likely than nondisabled people to report previous residence in a punitive institution (80.9 percent versus 74.5 percent), we estimated that disabled incarcerated people were more than twice as likely as nondisabled incarcerated people to have previously resided in a therapeutic institution (46.3 percent versus 19.6 percent) (exhibit 3).

The link between disability and previous residence in another institution was also stratified by race, ethnicity, and sex (exhibit 4). Among both men and women, disabled people were more likely than nondisabled people to report previous residence in any institution across most racial and ethnic groups. Still, the magnitude of the disparity in past institutional residence between disabled and nondisabled people varied across racial, ethnic, and sex groups. Further, Black and other race disabled men had the highest estimates of previous residence in another punitive institution—each more than 86 percent.

Disabled people were also more likely than nondisabled people to report previous residence in therapeutic institutions across all race, ethnicity, and sex categories. White disabled women had the highest estimate of previous residence in a therapeutic institution (64.9 percent).When we examined estimates of previous residence in therapeutic institutions, the gap between disabled and nondisabled people was greater among women than among men across all race and ethnicity categories. These sex disparities were not consistently observed for punitive or any institutions.

## Discussion

Despite growing concern about mass incarceration in the US, relatively little attention has been paid to the striking risks of incarceration and carceral punishment of disabled people. This study highlights how, as of 2016, disabled people were disproportionately incarcerated in state and federal prisons relative to nondisabled people and were also more likely to have previously resided in institutions that were punitive or traditionally considered therapeutic before their current incarceration. Previous studies have shown how destructive forces such as ableism, racism, and sexism work synergistically to jointly shape people’s incarceration risks and lives.^7–9^ Applying an intersectional lens, our study adds to the evidence base of the disproportionate burden that disabled people bear in carceral settings. By focusing attention on this unequal burden, we provide essential context for understanding the role of carceral institutions in shaping the lives and well-being of disabled people and producing, maintaining, and exacerbating disparities across a variety of outcomes.

A key takeaway from this study is the troubling overrepresentation of disabled people in state and federal prisons in the US. Although roughly 26 percent of the general population in the US was disabled as of 2018,^18^ we estimate that disabled people make up around two-thirds of the state and federal prison population, based on our 2016 findings. Consistent with evidence of racial, ethnic, and sex inequities in incarceration, our results demonstrate striking disparities at the intersections of disability, race, ethnicity, and sex. Although we found that disability prevalence was lower among Black and Hispanic incarcerated people compared with White incarcerated people, Black and Hispanic people were overrepresented in state and federal prisons in 2016. For example, Black people made up an estimated 33 percent of the state and federal prison population in our study despite being 14 percent of the US population, according to recent Census Bureau estimates.^19^ In contrast, White people were an estimated 31 percent of the state and federal prison population and 59 percent of the US population.^19^ Further, Black and Hispanic disabled men each make up less than 2 percent of the US population^19,20^ yet accounted for more than 18 percent and 12 percent of the state and federal prison population in our study population, respectively. In contrast, White nondisabled men were underrepresented in the state and federal prison population, making up around 23 percent of the US population^19,20^ but 7 percent of the state and federal prison population. Although women represented a smaller percentage of incarcerated people than men, incarcerated women had a higher prevalence of disability than incarcerated men, and within racial and ethnic groups, Black and Hispanic incarcerated women had especially high disability prevalence.

Our findings also provide new evidence of disparities in previous institutional residence at the intersections of disability, race, ethnicity, and sex. In considering the links between disability and incarceration, scholars and advocates increasingly call for consideration of the full spectrum of institutions that punish, confine, and isolate disabled people.^6–8^ Consistent with this idea, our findings reveal that compared with nondisabled incarcerated people, disabled incarcerated people were more likely to report previous residence in both punitive and therapeutic institutions. Although therapeutic institutions are intended to provide medical care and be more humane than punitive institutions, they often reinforce social control and structural oppression through mechanisms such as involuntary commitment.^9^ Disability is central to understanding how incarceration occurs through diverse institutional settings and comes to be portrayed as normal, expected, or even beneficial.^8^ Our findings reflect how diverse institutions can serve as sites of carceral logic—segregating, punishing, or controlling people—in ways that shape the lived experience of disabled people and serve as pathways to prison. Excluding more therapeutic institutions from studies of the criminal legal system risks masking the exclusionary mechanisms used to confine disabled people generally and disabled women in particular. Inattention to the full spectrum of carceral institutions in studies of disability limits understanding of the roles that both punitive and therapeutic institutions play in shaping the lives of disabled incarcerated people, and it further restricts policy and intervention efforts aimed at equity.

The disproportionate representation of disabled people in prisons in part reflects their heightened risks for incarceration. Our findings reveal that compared with nondisabled people, disabled people have high levels of contact with the criminal legal system. Disabled people also disproportionately experience socioeconomic disadvantage, including being unhoused and experiencing poverty, in ways that increase the risk for contact with law enforcement and the criminal legal system.^21^ Both medical and judicial institutions have played a role in the identification and treatment of disabled people in ways that link disability to notions of social danger, deviance, and criminality, ultimately contributing to high levels of confinement of disabled people in diverse institutional settings.^6–9^ Disability can be interpreted as a threat to law enforcement when people are unable to comply with commands that they cannot hear or physically respond to, leading to a greater probability of their being detained. More than 50 percent of disabled Black people have been arrested by age twenty-eight, suggesting that the criminal legal system is disproportionately surveilling this population.^22^ Not only are disabled people more likely to be policed, but protection conferred through the Americans with Disabilities Act of 1990 during arrest is legally ambiguous,^23^ rendering disabled people especially vulnerable in their interactions with the criminal legal system. Postarrest, significant discretion is left to judges, who make critical decisions that shape sentence length, reincarceration, and supervision after release. Although there are legally mandated accommodations for disabled people, they are inconsistently granted, with far-reaching consequences.^23^ For example, 45 percent of state prison admissions in the US are from probation or parole violations.^24^ Without legal protections such as accessible transportation, it can be difficult for disabled people to comply with mandates, contributing to high risks for rearrest and reincarceration.^23^ These heightened risks of incarceration among disabled people are exacerbated for racially minoritized people, further highlighting the highly unequal and discriminatory nature of policing, carceral control, and punitive punishment in the US.

Connecting disabled people to appropriate and accessible community resources can reduce incarceration risks among them.

In addition to the high risks of incarceration experienced by disabled people, being incarcerated can increase disablement and exacerbate existing disability. The physical and social environments of prisons and jails, characterized by high levels of stress and violence, are disabling.^10,11^ Incarceration is associated with excess mortality that translates to reduced life expectancy.^25^ The association between incarceration and mortality may be more profound for disabled people, who are disproportionately exposed to practices that harm health and who face especially long sentences. Solitary confinement is particularly disabling, especially when used for extended periods.^26^ Prisons disproportionately segregate disabled people for many reasons, including the unavailability of cells that can accommodate physically disabled people. In addition, prisons often fail to provide necessary accommodations or assistive devices such as access to interpreters or mobility aids.^26^ These practices profoundly harm disabled people psychologically and can also reduce their access to medical therapies and required care assistance.^26^ Trauma before and during incarceration differentially contributes to the disabling nature of carceral institutions for women, especially Black women. A study published in 2016 found that more than 80 percent of incarcerated women had experienced violence prior to arrest and that incarcerated women were more likely than incarcerated men to be assaulted inside carceral institutions.^27^ This exposure is associated with high rates of psychiatric disability and may increase risks for incarceration.^27^

## Policy Implications

Our results highlight the importance of centering disabled people in policy and political actions aimed at reducing imprisonment and improving the well-being of people while incarcerated and postrelease. Such a policy agenda must be attuned to the many institutions, both punitive and therapeutic, that make up the carceral system, as well as to the intersectional inequalities maintained, generated, and exacerbated by carceral institutions. As a first step, better data on the prevalence of disability in carceral spaces are needed, as are longitudinal data on the well-being and needs of disabled people in prisons, jails, and other carceral institutions. Longitudinal administrative and survey data on incarcerated people, including disabled incarcerated people, will facilitate future research aimed at improving understanding of the unique life-course experiences, needs, and challenges facing this population in ways that can inform policy and intervention.

More important, our results underscore the urgent need to prevent and redress the disproportionate incarceration of disabled people. Efforts to decarcerate will reduce the social, political, and legal exclusion of disabled people. Policies that support the social and financial well-being of disabled people, including those that expand health care access, improve community care, expand the social safety net, and fund public health infrastructure, are likely to reduce imprisonment and reincarceration rates among disabled people. Connecting disabled people to appropriate and accessible community resources, including employment, housing, and holistic care management, can also reduce incarceration risks among them.

Disabled people may have unique health needs while incarcerated. Prisons are one of the only institutions in the US where health care access is guaranteed by law; however, this care is often outsourced or subcontracted to for-profit organizations, and its quality varies. Regulations to improve the accountability of prisons to provide comprehensive and quality care are needed, including clinical independence for providers, publishing aggregate information about health outcomes, establishing protected complaint mechanisms, and monitoring by independent organizations. These terms are commonplace in other US health care institutions, guaranteed in other countries, and articulated in United Nations documents including the Standard Minimum Rules for the Treatment of Prisoners.^28^ Implementing models of care such as the medical home, which co-locates care led by one clinician, is another strategy that can improve care, especially for disabled people who may receive care from multiple providers. Given the expansive network of prisons in the US, mandating accreditation for medical care in prisons is warranted in an effort to increase safety levels, improve the process of care, and promote better clinical outcomes.^29^

Policy makers should also consider the specific needs of disabled people postrelease. Many social benefits are terminated when a person enters the carceral system, including health insurance, Supplemental Security Income, and Social Security Disability Insurance payments. Medicaid eligibility for returning citizens varies by state, and reapplying for these services is seldom facilitated by carceral institutions. Instituting automatic eligibility and renewal in Medicaid on release would help ensure that disabled people have guaranteed access to care as they reintegrate into society. Similarly, special enrollment opportunities should be implemented so that formerly incarcerated disabled people and older adults can enroll in Medicare and prevent delays or gaps in coverage postrelease. Transferring care to the community requires discharge planning, and disabled people are disproportionately served by institutions that seldomly coordinate to share medical records, make first appointments, and ensure that prescriptions are available after release. This may be even more complex for disabled people without proper forms of identification and supportive social networks after extended periods of incarceration. Providing continuity of care between prison services and community providers on release can promote self-efficacy and community integration. This process may be best facilitated by navigators who have been previously incarcerated themselves. Policies that fund interventions on release can reduce reentry into carceral institutions, especially for disabled people receiving health care services.^30^

## Conclusion

Disability is a neglected axis of inequality in the carceral system. Still, our findings indicate that disabled people have been overrepresented in US prisons and other carceral institutions, with inequities by race, ethnicity, and sex. Health policy experts, practitioners, and advocates must be attuned to the roles that carceral surveillance, control, and punishment play in the lives and well-being of disabled people and work to redress inequities sustained by the carceral system.■

## Supplementary Material

supplement

## Figures and Tables

**EXHIBIT 1:**
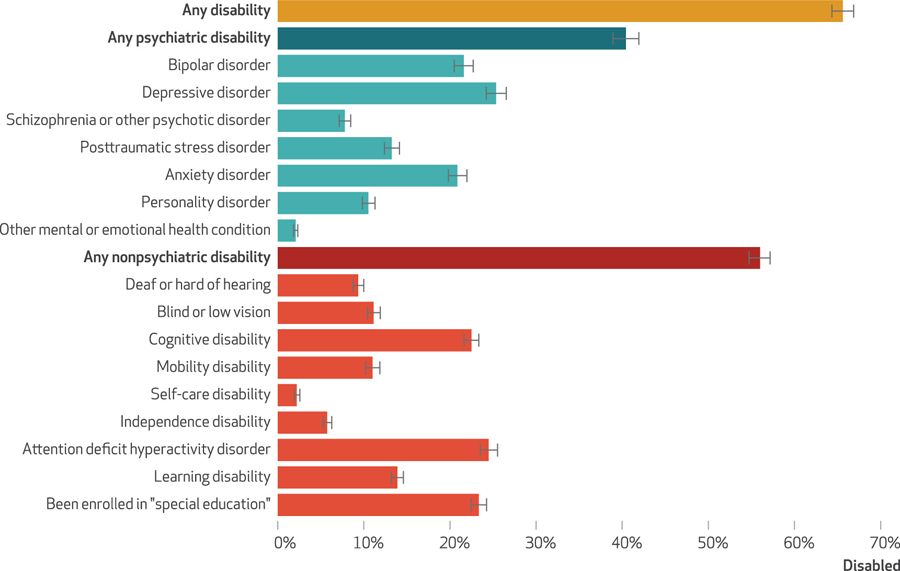
Disability prevalence among people incarcerated in state or federal prisons in the US, by disability type, 2016 **source** Authors’ analysis of data from the Bureau of Justice Statistics, Survey of Prison Inmates (SPI), 2016. **notes** *N* = 22,660. Estimates reflect the proportion of people in state and federal prisons who reported various disability types. The “any disability” category includes anyone who reported either a psychiatric or a nonpsychiatric disability. The SPI survey questions are in the appendix (see note 17 in text).

**EXHIBIT 2:**
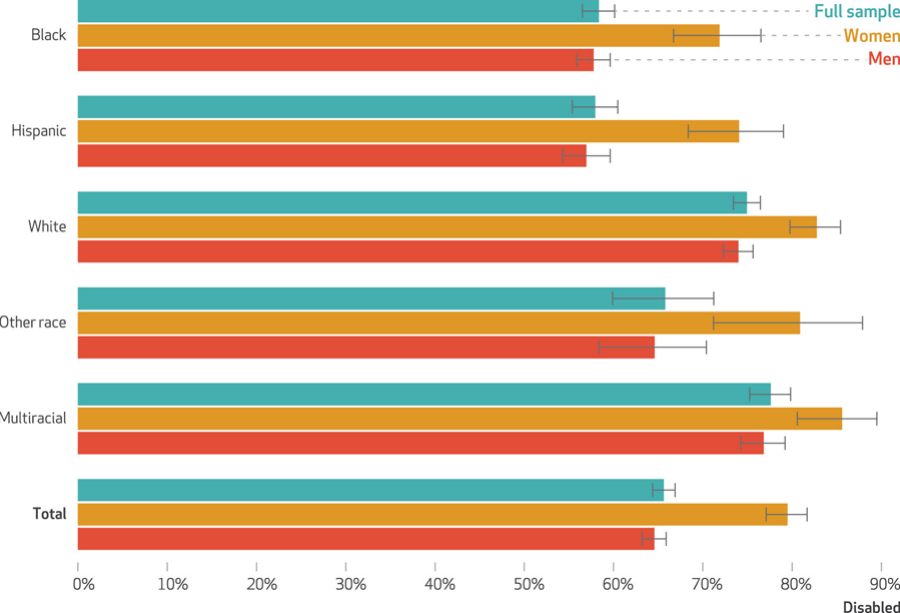
Disparities in disability status among people incarcerated in state or federal prisons in the US, by race, ethnicity, and sex, 2016 **source** Authors*’* analysis of data from the Bureau of Justice Statistics, Survey of Prison Inmates (SPI), 2016. **notes** *N* = 22,660. Estimates reflect the proportion of people with any disability. Estimates are stratified by race and ethnicity. For each racial and ethnic group, we estimate the prevalence of disability among the full sample, among women, and among men. The race and ethnicity variable is constructed by the SPI using respondents*’* self-identified race and Hispanic ethnicity. The 5 categories are non-Hispanic Black, Hispanic, non-Hispanic White, other single race and non-Hispanic, and multiracial and non-Hispanic.

**EXHIBIT 3:**
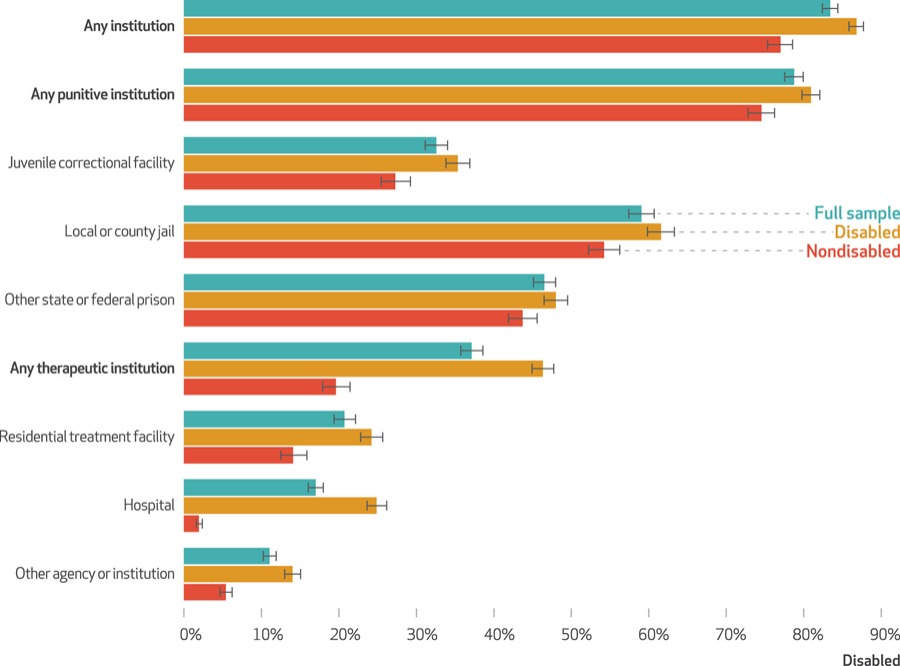
Previous residence in other institutions before current incarceration among people incarcerated in state or federal prisons in the US, by disability status, 2016 **source** Authors*’* analysis of data from the Bureau of Justice Statistics, Survey of Prison Inmates, 2016. **notes** *N* = 22,660. Estimates reflect the percent of people who had previously resided in another institution before their current incarceration. We consider someone to have previously resided in “any institution” if they reported previous residence in any punitive or therapeutic institution. “Any punitive institution” includes those who previously resided in a juvenile correctional facility, local or county jail, or other state or federal prison. “Any therapeutic institution” includes those who previously resided in a residential treatment facility for alcohol or drug use; hospital for mental health treatment; or other agency or institution, such as a mental health facility or group home. Estimates are stratified by disability status. People are considered disabled if they reported any disability (psychiatric or nonpsychiatric). People are considered nondisabled if they reported neither psychiatric nor nonpsychiatric disability.

**EXHIBIT 4:** Previous residence in other institutions before current incarceration among incarcerated people in the US, by disability, race, ethnicity, and sex, 2016

	Any institution		Punitive institution		Therapeutic institution	
	Disabled	Nondisabled	Disabled	Nondisabled	Disabled	Nondisabled
**WOMEN**						
Black	82.45%	65.69%****	74.69%	62.30%**	48.23%	18.97%****
Hispanic	84.28	69.49****	73.28	65.93*	52.25	23.83****
White	85.65	74.26****	71.52	62.92***	64.92	40.91****
Other race	85.57	72.59	81.09	70.78	58.00	32.46*
Multiracial	88.96	78.30*	74.93	69.54	62.38	27.10****
**MEN**						
Black	89.35%	80.88%****	86.14%	79.50%****	38.99%	16.97%****
Hispanic	85.10	72.62****	80.88	71.17****	37.90	12.07****
White	85.15	75.44****	77.73	70.01****	52.76	31.10****
Other race	88.90	70.70***	86.73	69.50***	47.61	22.91***
Multiracial	88.42	82.19***	81.84	81.45	50.51	22.17****

**source** Authors*’* analysis of data from the Bureau of Justice Statistics, Survey of Prison Inmates (SPI), 2016. **notes** *N* = 22,660. Estimates reflect the percent of people who had previously resided in another institution before their current incarceration, stratified by disability, race, ethnicity, and sex. We consider someone to have previously resided in “any institution” if they reported previous residence in any punitive or therapeutic institution; these institutions are listed in the notes to exhibit 3. Definitions of disabled and nondisabled people are in the notes to exhibit 1. The race and ethnicity variable is constructed by the SPI using respondents*’* self-identified race and Hispanic ethnicity. The 5 categories are described in the notes to exhibit 2.

* *p* < 0.10

** *p* < 0.05

*** *p* < 0.01

**** *p* < 0.001
