# Supplementary material for: The Links Between Disability, Incarceration, And Social Exclusion
Source: Health Aff (Millwood). Author manuscript; Available in PMC 2023 Oct 1. (PMC10335036; doi:10.1377/hlthaff.2022.00495)
Supplement: supplement [file NIHMS1904294-supplement-supplement.pdf]

APPENDIX

Survey of Prison Inmates, 2016

DISABILITY QUESTIONS:

Psychiatric disabilities:

(1) *Bipolar disorder:* "Have you ever been told by a medical doctor or a mental health professional, such as a psychiatrist or psychologist that you had manic depression, a bipolar disorder, or mania?"

(2) *Depressive disorder:* "Have you ever been told by a medical doctor or a mental health professional, such as a psychiatrist or psychologist that you had a depressive disorder?"

(3) *Schizophrenia/other psychotic disorder:* "Have you ever been told by a medical doctor or a mental health professional, such as a psychiatrist or psychologist that you had schizophrenia or another psychotic disorder?"

(4) *Post-traumatic stress disorder (PTSD):* "Have you ever been told by a medical doctor or a mental health professional, such as a psychiatrist or psychologist that you had post-traumatic stress disorder, also known as PTSD?"

(5) *Anxiety disorder:* "Have you ever been told by a medical doctor or a mental health professional, such as a psychiatrist or psychologist that you had an anxiety disorder, such as panic disorder or obsessive-compulsive disorder, also known as OCD?"

(6) *Personality disorder:* "Have you ever been told by a medical doctor or a mental health professional, such as a psychiatrist or psychologist that you had a personality disorder, such as antisocial or borderline personality?"

(7) *Other mental/emotional health condition:* "Have you ever been told by a medical doctor or a mental health professional, such as a psychiatrist or psychologist that you had or other mental or emotional health condition?"

Non-psychiatric disabilities:

(1) *Deaf or hard of hearing:* "Are you deaf or do you have serious difficulty hearing?"

(2) *Blind or low vision:* "Are you blind or do you have serious difficulty seeing even when wearing glasses?"

(3) *Cognitive disability:* “Because of a physical, mental, or emotional problem, do you have serious difficulty concentrating, remembering, or making decisions?”

(4) *Mobility disability:* “Do you have serious difficulty walking or climbing stairs?”

(5) *Self-care disability:* “Do you have difficulty dressing or bathing?”

(6) *Independence disability:* “Because of a physical, mental, or emotional problem, do you have difficulty doing activities on your own such as going to meal time, going outside, working in or outside of this facility, going to classes, or attending programs?”

(7) *Attention deficit hyperactivity disorder:* “Has a doctor, psychologist, or teacher ever told you that you have an attention deficit disorder, sometimes call ADD or ADHD?”

(8) *Learning disability:* “Has a doctor, psychologist, or teacher ever told you that you have a learning disability, such as dyslexia or dyscalculia?”

(9) *Enrolled in special education:* “Have you ever been enrolled in special education classes, sometimes called S-P-E-D?”

APPENDIX EXHIBIT:

Composition of State and Federal Prison Population: Disability, Race and Ethnicity, and Sex, United States, 2016

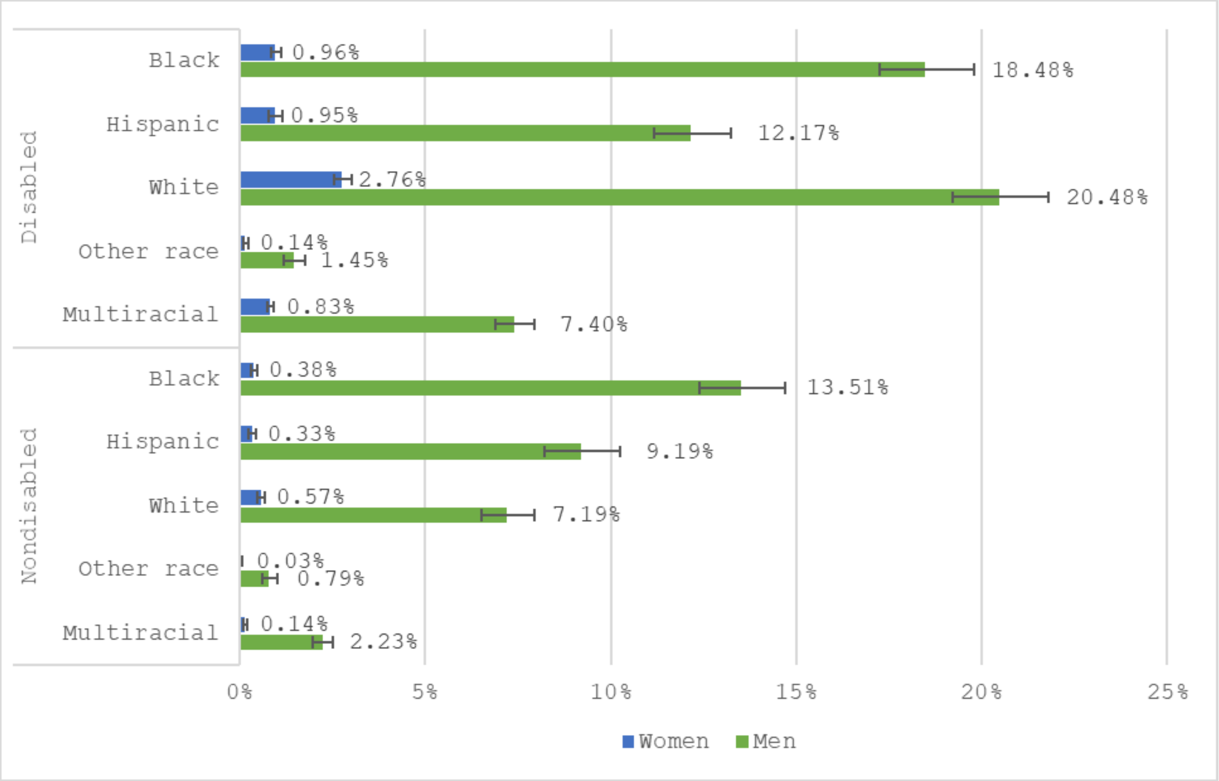

SOURCE: Authors' analysis of data from the Bureau of Justice Statistics, Survey of Prison Inmates, 2016. (N= 22,660).

NOTES: The data reflect the composition of the state and federal prison population at the intersection of disability, race and ethnicity, and sex. People are considered disabled if they report any disability (psychiatric or non-psychiatric). People are considered nondisabled if they report neither psychiatric nor non-psychiatric disability. The race and ethnicity variable is constructed by the Survey of Prison Inmates, using respondents' self-identified race and Hispanic ethnicity. The 5 categories are non-Hispanic Black, Hispanic, non-Hispanic White, non-Hispanic other single race, and non-Hispanic multiracial.
